# Metagenomic insights into pathogen diversity and public health risk of *Haemaphysalis longicornis* in Gansu Province of China

**DOI:** 10.1371/journal.pntd.0014713

**Published:** 2026-09-15

**Authors:** Biao Yang, Qiaoyun Ren, Junwei Wang, Xinxin Zhang, Hong Yin, Jinming Wang, Guiquan Guan

**Affiliations:** 1 State Key Laboratory of Animal Disease Control and Prevention, Key Laboratory of Veterinary Parasitology of Gansu Province, Lanzhou Veterinary Research Institute, Chinese Academy of Agricultural Sciences, Lanzhou, China; 2 Jiangsu Co-Innovation Center for the Prevention and Control of Important Animal Infectious Disease and Zoonoses, Yangzhou University, Yangzhou, China; KALRO: Kenya Agricultural and Livestock Research Organization, KENYA

## Abstract

The tick *Haemaphysalis longicornis* vectors numerous zoonotic pathogens, yet its pathogen diversity and associated spatial health risks are not fully characterized. To profile this vector, we performed metagenomic sequencing on 290 *H. longicornis* ticks across 29 sites in Gansu Province, China. We resolved 25 bacterial and one protozoan genera, with computational analyses revealing population-level distribution patterns and identifying environmental factors as key drivers of spatial variation.We detected marker genes associated with 12 pathogenic microorganisms. Alongside established threats (*Rickettsia conorii*, *R. rickettsii*, *Coxiella burnetii*, and *Francisella novicida*), we additionally identified several pathogens not previously reported in this vector, including *Candidatus Bartonella merieuxii*, *Acinetobacter lwoffii*, *Shigella flexneri*, *Staphylococcus chromogenes*, *Moraxella atlantae*, *Pseudomonas alabamensis*, and *Trypanosoma spp*. Furthermore, four potentially novel strains were identified within *R. conorii*, *Escherichia coli*, and *Acinetobacter pseudolwoffii*. Spatial Bayesian risk modeling localized the highest exposure risks to the Linxia, Zhangye, and Wuwei regions. The tick microbiome contained 31 clinically prioritized antibiotic resistance genes (ARGs), many of which co-localized with mobile genetic elements (MGEs). Overall, these findings reveal the diversity of pathogens associated with *H. longicornis* and their public health relevance, supporting future efforts in disease surveillance and risk assessment.

## Author summary

Ticks can carry microorganisms that affect human and animal health, but the diversity of these microorganisms in *Haemaphysalis longicornis* remains poorly characterized in northwestern China. We collected 290 ticks from 29 locations in Gansu Province and used DNA-based screening to investigate microorganisms of possible medical or veterinary relevance and antibiotic resistance genes. We detected DNA from 12 potentially important microorganisms. Several detections may represent the first records in this tick species, and four divergent genetic lineages require further taxonomic investigation. Detection patterns differed among locations and were statistically associated with local environmental variables. Spatial analyses highlighted Linxia, Zhangye, and Wuwei as priorities for additional sampling and surveillance. We also identified 31 types of antibiotic resistance genes. Some occurred on the same DNA fragments as putative mobile genetic elements, suggesting, but not demonstrating, the potential mobility of these resistance genes. These results provide a baseline for understanding tick-associated microorganisms and resistance genes in Gansu Province. The findings also highlight specific microorganisms and locations that warrant further investigation through larger-scale surveys and targeted laboratory confirmation.

## Introduction

*Haemaphysalis longicornis* is a hematophagous ectoparasite whose different developmental stages can infest a wide range of vertebrate hosts, including rodents, domestic animals, and certain bird species [1,2]. Native to Asia, this tick species is widely distributed across China, Korea, Japan, Russia, and India [3–5]. In China, it has been reported in all 32 provinces, autonomous regions, and municipalities, making it one of the most prevalent and epidemiologically significant tick species nationwide [6]. To date, at least 30 human pathogens have been associated with *H. longicornis*, including species of *Rickettsia*, *Anaplasma*, *Ehrlichia*, *Borrelia*, *Babesia*, *Theileria*, *Francisella*, *Coxiella*, as well as various viruses [7–9]. In recent years, an increasing number of pathogens linked to *H. longicornis* have been identified worldwide [10–14], and this tick has also been recognized as a potential reservoir and vector for antibiotic resistance genes (ARGs) [15,16]. These reports underscore the critical importance of *H. longicornis* in public health surveillance and risk assessment.

However, our current understanding of the geographic distribution, diversity of transmitted pathogens, and transmission risk of pathogens carried by *H. longicornis* remains limited. Such knowledge is essential for elucidating the interactions among pathogens, vectors, hosts, and environmental factors, as well as for improving risk assessment frameworks. Gansu province, located in the northwest of China (92°13′–108°46′ E, 32°11′–42°57′ N), lies in the convergence zone of Loess Plateau, Qinghai-Tibet Plateau and Inner Mongolian Plateau. It has various geomorphic features, such as mountain land, highland, grassland, plain, valley, desert and gobi, and 4 types of climates, subtropical continental monsoon climate, temperate continental monsoon climate, temperate continental arid climate and plateau mountain climate [17]. The diverse features of climate and geography provide the ecological niche for different tick species, and more than 33 tick species have been identified in Gansu Province [18]. The diversity of climate, geography and tick species of Gansu province is a suitable natural model for performing the study of tick-borne pathogen diversity and risk assessment in China.

In this study, we conducted a metagenomic survey of *H. longicornis* collected from Gansu Province, China, to characterize the diversity and distribution patterns of associated pathogens. In addition, we performed a model-based comprehensive assessment of the public health risks posed by this tick species and further investigated its antibiotic resistance genes (ARGs). Together, these analyses aim to provide a better understanding of the potential threats posed by *H. longicornis* to public health and veterinary health in the region.

## Materials and methods

### Ethics statement

Tick collection from livestock was approved by the Animal Ethics Committee of the Lanzhou Veterinary Research Institute, Chinese Academy of Agricultural Sciences. All sampling procedures were conducted in accordance with the Animal Ethics Procedures and Guidelines of the People’s Republic of China (Permit No. LVRIAEC-2022–001). Verbal informed consent was obtained from the owners of all animals prior to sampling.

### Sample collection

This cross-sectional study was conducted between April and June 2025. During this period, a total of 290 parasitic ticks were collected from 29 sampling sites across all 12 prefecture-level cities in Gansu Province, China. Sampling sites were selected to represent diverse ecological environments, including grasslands, mountainous regions, hills, deserts, and Gobi areas. Ticks were directly removed from cattle, sheep, and camels using sterile forceps, and the geographic coordinates (latitude and longitude) of each sampling site were recorded (S1 Table). To evaluate microbial transmission across generations, engorged female ticks collected from the field were maintained under laboratory conditions for oviposition and subsequent offspring development. The resulting offspring ticks were collected and subjected to microbiome sequencing together with their corresponding parental ticks. Environmental factor data were obtained from the National Geomatics Center of China (https://www.ngcc.cn/). All samples were preserved in portable refrigeration containers with dry ice and subsequently transferred to a −80 °C freezer.

### Nucleic acid extraction and total DNA sequencing

All field-collected tick samples were thoroughly surface-sterilized by washing twice with 75% ethanol for 30 s each. Ticks were pooled according to sampling sites, and total DNA was extracted from each pooled sample. Each sequencing library was constructed using DNA pooled from ten individual ticks. Library construction was performed using the NEBNext Ultra II DNA Library Prep Kit (New England Biolabs, Ipswich, MA, USA). After adaptor ligation, libraries were enriched by 10 cycles of PCR amplification. All libraries were pooled in equimolar concentrations, denatured, diluted to the optimal loading concentration, and sequenced on the Illumina NovaSeq 6000 platform (Illumina, San Diego, CA, USA), generating 150 bp paired-end reads.

### Genome assembly and microbiome identification

Raw sequencing reads were filtered using fastp (v1.0.1) [19] by removing reads containing adapters, reads with an average quality score below Q20, reads containing more than 10% ambiguous bases (N), and reads shorter than 50 bp. The clean reads from the Metagenomics libraries were mapped to the SILVA rRNA database (release 132) [20] using Bowtie 2 (version 2.5.2) to remove rRNA-related and host reads [21]. De novo assembly of clean reads was performed using MEGAHIT (v1.2.9) [22]. Contigs shorter than 500 bp were excluded prior to statistical analysis and gene prediction. Open reading frames (ORFs) were predicted from scaftigs ≥ 500 bp in each sample using MetaGeneMark (v3.26) [23]. Redundant ORFs were then removed using CD-HIT (v4.8.1) [24] to generate a non-redundant initial gene catalog. Clean reads from each sample were subsequently aligned to the gene catalog using Bowtie2, and the number of mapped reads per gene was calculated. Genes with ≤ 2 reads in each sample were excluded, and the remaining genes were defined as unigenes for subsequent analyses. Taxonomic annotation was conducted by aligning Unigenes against microbial sequences (Bacteria, Fungi, Archaea, and Viruses) extracted from the NCBI non-redundant (NR) protein database using DIAMOND (v2.1.16) [25]. A significant hit was defined by an E-value smaller than 1  ×  10^−5^. Given that individual sequences may yield multiple hits, taxonomic assignments were performed using the Lowest Common Ancestor (LCA) algorithm implemented in MEGAN (v6.25.10) [26].

### 16S rRNA reconstruction and strain-level phylogenetic analysis of pathogens

Bacterial 16S rRNA genes were extracted from the MEGAHIT-assembled metagenomic contigs and used to construct a reference index with Bowtie2. Clean reads were subsequently mapped to this index for sequence alignment. The resulting alignment files were processed and converted using SAMtools (v1.23) [27]. The mapped reads were subsequently extracted and reconstructed into 16S rRNA gene sequences for bacterial taxa. Phylogenetic trees were then inferred using IQ-TREE (v3.0.1) [28] based on the maximum likelihood method, with 1,000 ultrafast bootstrap replicates performed to assess branch support.

For strain-level identification, a custom reference database was built using StrainScan [29], and sequencing data were queried against this database to determine candidate strains. Corresponding reference genomes were subsequently retrieved from the NCBI RefSeq database (https://www.ncbi.nlm.nih.gov/datasets/genome/), including *Acinetobacter pseudolwoffii* (GCF_002803605.1), *Rickettsia raoultii* (GCF_000940955.1), *Rickettsia conorii* (GCF_048495935.1), and *Escherichia coli* (GCF_002056145.1). Clean reads were mapped to these reference genomes for downstream analyses, and single nucleotide polymorphisms (SNPs) were identified using BCFtools (v1.20) [27]. Variant calling was performed with bcftools call, and low-confidence variants were filtered using thresholds of QUAL < 30 and sequencing depth (DP) < 10. Consensus genome sequences were then generated from the filtered variants using bcftools consensus. To evaluate genomic similarity, average nucleotide identity (ANI) between reconstructed consensus genomes and reference genomes was calculated using FastANI (v1.34) [30]. For phylogenetic analysis, core-genome alignments were generated using Parsnp (Harvest suite). Here, the core genome was defined as the genomic regions conserved and shared across all genomes included in the analysis. Full-length core SNP alignments were subsequently extracted using HarvestTools [31]. Maximum likelihood phylogenetic trees were subsequently inferred using IQ-TREE under the best-fit substitution model selected by ModelFinder (MFP), with branch support assessed using 1,000 ultrafast bootstrap replicates and 1,000 SH-aLRT tests.

### Data processing and statistical analysis

Statistical analyses were performed using Python (version 3.10.11). We evaluated the consistency in microbial community composition and abundance between parental and offspring samples using Bray–Curtis similarity indices and Pearson correlation coefficients. Environmental variables, including normalized difference vegetation index (NDVI), population density (Pop_density), and livestock density (Livestock_density), were obtained for each sampling location from the Resource and Environmental Science Data Center (https://www.resdc.cn/) and the National Bureau of Statistics of China (https://www.stats.gov.cn/). The spatial distributions of these variables were visualized in three-dimensional space using the Axes3D function from the mpl_toolkits.mplot3d module of Matplotlib (version 3.10.7) [32], a Python plotting library. To quantitatively evaluate the effects of environmental factors on the spatial distribution of tick-borne pathogenic genera, ridge regression implemented in the scikit-learn library [33] was applied to estimate the linear relationships between environmental variables and pathogen abundance. Bootstrap resampling (n = 1000) was used to calculate 95% confidence interval (CI) and p-values for regression coefficients. Statistical significance was defined as p < 0.05.

We used an integrated spatial risk assessment framework to quantify the distribution of zoonotic pathogens and their associated public health risks. Pathogen abundance data were weighted according to the relative pathogenic importance of detected microorganisms and used to calculate the Shannon diversity index for each sampling location. Environmental and socioeconomic variables were subsequently standardized to construct composite indices of exposure (human population density and livestock abundance) and vulnerability (normalized difference vegetation index NDVI, gross domestic product GDP, and healthcare capacity). Spatial relationships among sampling sites were modeled using an inverse distance weighting (IDW) matrix, and global spatial autocorrelation was assessed using Moran’s I. To mitigate subjective bias in parameterizing the multi-dimensional risk drivers (hazard, exposure, and vulnerability), an information entropy weighting method was integrated to elicit objective priors. The derived entropy weights were scaled and employed as the concentration parameter α for the Dirichlet prior distribution.The comprehensive pathogen risk was modeled by superimposing spatial spillover effects onto localized drivers. The baseline localized risk (LocalRisk) [34,35] was defined as:


$$\begin{array}{c} \text{LocalRis}{\text{k}}_{\text{i}}={\text{w}}_{\text{h}}\cdot \text{Hazar}{\text{d}}_{\text{i}}+{\text{w}}_{\text{e}}\cdot \text{Exposur}{\text{e}}_{\text{i}}+{\text{w}}_{\text{v}}\cdot \text{Vulnerabilit}{\text{y}}_{\text{i}} \end{array}$$

where ${\text{w}}_{\text{h}},{\text{w}}_{\text{e}},{\text{w}}_{\text{v}}\sim \text{Dirichlet}(\alpha )$ are the posterior risk driver weights. To rigorously account for the theoretical transmission network and unobserved spatial spillovers, a Spatial Autoregressive (SAR) process was formulated:


$$\begin{array}{c} \text{Risk}=(\text{I}-\rho \text{W}{)}^{-\text{1}}\cdot \text{LocalRisk} \end{array}$$

where ρ~Beta(2,5) represents the spatial autoregressive coefficient, quantifying the infectious diffusion rate. The model was sampled using the No-U-Turn Sampler (NUTS) via Markov Chain Monte Carlo (MCMC) simulations, with 1,000 tuning (burn-in) steps followed by 2,000 posterior draws across 2 chains, to derive posterior risk means and 95% Highest-Density Intervals (HDI).

### Annotation of antibiotic resistance genes (ARGs) and mobile genetic elements (MGEs)

Open reading frames were predicted from Unigenes using Prodigal (v2.6.3) [36]. Predicted ORFs subsequently were aligned against the ResFinder ARG database [37] using DIAMOND to identify antibiotic resistance genes (ARGs). Reads were mapped back to annotated ORFs using Bowtie2, and SAMtools was used to calculate reads per kilobase per million mapped reads (RPKM) values to estimate the relative abundance of each ARG. To identify mobile genetic elements (MGEs), nucleotide sequences were aligned against the TnCentral, ISfinder, and INTEGRALL databases using BLASTN (v2.15.0) [38–41]. The ARGs identified were filtered based on criteria of 60% alignment coverage and 70% amino acid identity to the reference sequence, and the MGEs were filtered based on 50% alignment coverage and 70% nucleotide identity to the reference sequence.

## Results

### Metagenomic survey of *Haemaphysalis longicornis*

**Fig 1:**
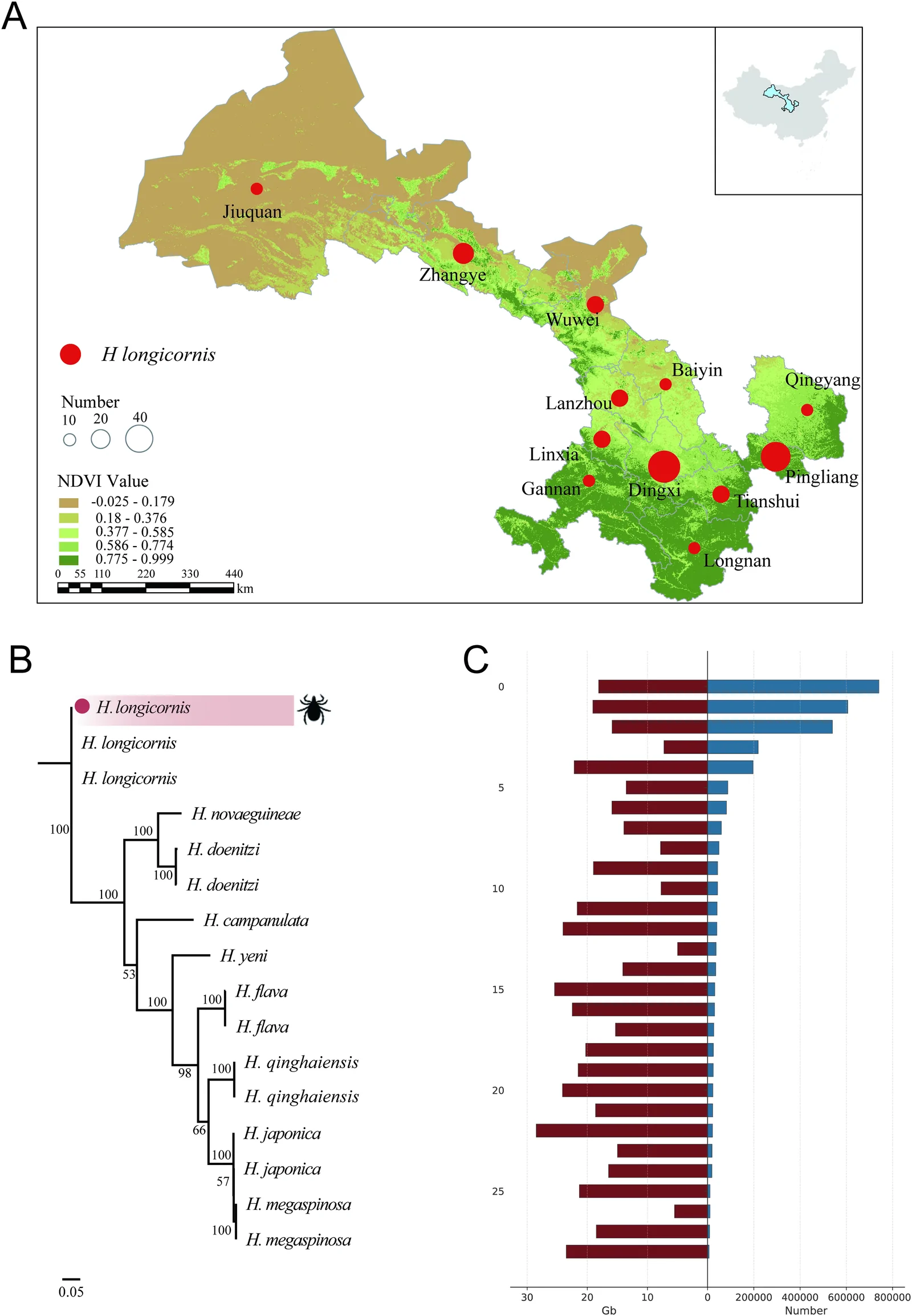
Geographic distribution of *Haemaphysalis longicornis* sampling sites in Gansu Province, China, and basic sequencing statistics. Map source: The basemap was obtained from Natural Earth (https://www.naturalearthdata.com/downloads/10m-cultural-vectors/). Sampling locations were plotted by the authors. **(A)** Geographic distribution of sampling sites. Circle size indicates the number of tick samples collected in each region. **(B)**. Maximum-likelihood phylogenetic tree based on the mitochondrial cytochrome c oxidase subunit I (COXI) gene of *H. longicornis*. **(C)** Distribution of sequencing throughput (in gigabases, Gb) and assembly metrics across metagenomic libraries.

A total of 290 ticks were collected from 12 prefecture-level cities, including Dingxi (n = 70), Lanzhou (n = 20), Pingliang (n = 60), Gannan (n = 10), Zhangye (n = 30), Baiyin (n = 10), Linxia (n = 20), Jiuquan (n = 10), Longnan (n = 10), Tianshui (n = 20), Qingyang (n = 10), and Wuwei (n = 20). Sampling sites covered a wide range of land-use types across the province, including grasslands, mountainous areas, hills, deserts, and Gobi regions (Fig 1A). All ticks were identified as *H. longicornis* based on morphological characteristics and cytochrome c oxidase subunit I (COXI) gene sequence comparison (Fig 1B). The 290 individual ticks were grouped according to collection sites and pooled into 29 sequencing libraries, with each library comprising 10 ticks. Metagenomic sequencing generated approximately 501.88 Gb of raw data in total, and the number of assembled contigs per library ranged from 6,448–740,899 (Fig 1C). Libraries with relatively high sequencing depth but fewer assembled contigs were further examined, and the reduced assembly output was mainly attributed to a high proportion of host-derived reads, resulting in fewer microbial sequences available for assembly.

### Taxonomic annotation of bacterial genera and their abundance patterns in *H. longicornis*

**Fig 2:**
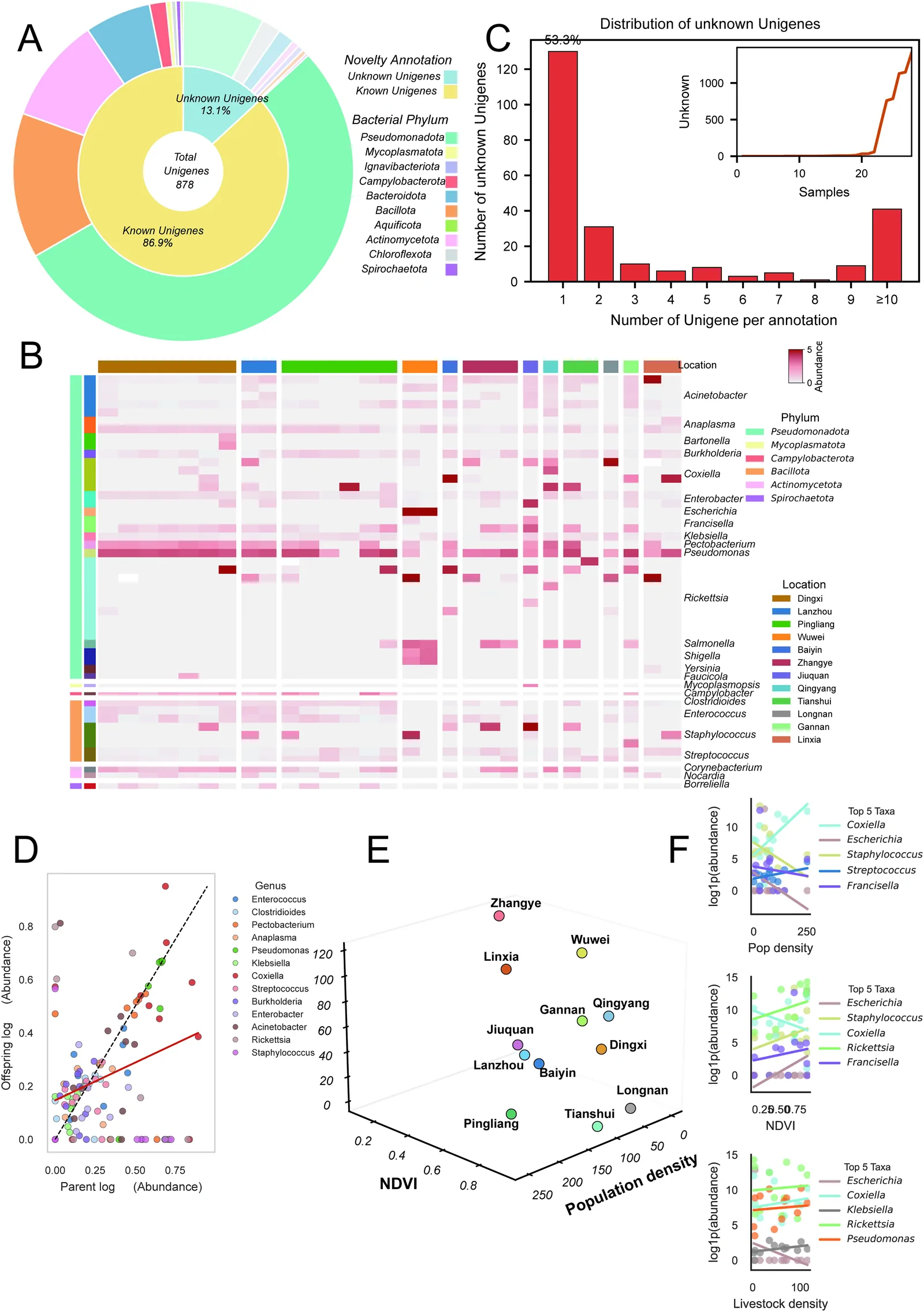
Bacterial genus composition and distribution in *H. longicornis.* **(A)**. Proportions of known and unknown bacterial Unigenes and their distribution at the phylum level. **(B)**. Relative abundance of bacteria genus detected in each library. Each cell in the heat map represents the normalized number of reads belonging to the bacteria genus. **(C)**. Distribution of Unigene counts and accumulation curves of unclassified bacterial taxa. The outer bar chart shows the distribution of annotated Unigene counts and the inner curve represents the accumulation of Unigenes that do not describe bacterial species. **(D)** Relationships between bacterial genus abundances in F and F1 samples. In the scatter plot, different colored points represent different bacterial genera, and the dashed line indicates a Pearson correlation coefficient of 1. **(E)** Spatial distribution of sampling locations along normalized difference vegetation index (NDVI), population density (Pop-density), and livestock density (Livestock-density) gradients. **(F)** Ridge regression–based trends illustrating the top five responses of bacterial genera to natural and socio-economic environmental variables.

By integrating all sequencing data, we applied a de novo metagenomic assembly pipeline and obtained a total of 1,069,708 unigenes. Taxonomic annotation was subsequently performed for the assembled unigenes in each library. In total, 878 unigenes were assigned to the bacterial species level, of which 86.9% were classified into known bacterial taxa, while 13.1% remained unclassified (Fig 2A). A total of 10 bacterial phyla were identified, with *Pseudomonadota* dominating the bacterial community (63.85%). At the genus level, 25 bacterial genera were detected, including a range of potentially pathogenic microorganisms. Among these, *Rickettsia* showed the highest relative abundance (10.00%), followed by Coxiella (8.80%), *Acinetobacter* (7.13%), *Francisella* (4.45%), and *Escherichia* (2.01%) (Fig 2B). Unigene accumulation curve analysis indicated that the discovery rate of bacterial taxa had not yet reached saturation, suggesting extensive microbial diversity within *H. longicornis* and the presence of a substantial number of yet-unresolved bacterial taxa (Fig 2C).

We applied Bray–Curtis similarity analysis to assess the stability and transmission of bacterial communities between parental ticks collected from the field and their laboratory-reared offspring. High community similarity was observed in samples from Dingxi (DX1-DX3), Lanzhou (LZ), and Pingliang (PL1), with values ranging from 0.76 to 0.93. In contrast, several sample pairs, including those from Wuwei (WW), exhibited markedly lower similarity (down to 0.24), suggesting potential local divergence driven by environmental or stochastic factors. At the genus level, Pearson correlation analysis of relative abundances between parental and offspring samples revealed moderate correlations (e.g., *Enterococcus*, r = 0.78; *Rickettsia*, r = -0.27); however, none remained statistically significant after false discovery rate correction (Fig 2D). This lack of significant linear association likely reflects substantial intergenerational fluctuations in specific taxa.

In the ridge regression analysis, the effects of environmental variables on the abundance of selected pathogenic genera were evaluated (Fig 2E). The results showed that the abundances of tick-associated pathogens *Coxiella* and *Rickettsia* were primarily driven by population density and vegetation coverage. Specifically, *Coxiella* abundance was significantly higher in densely populated areas (β = 1.99, 95% confidence interval [CI] = 0.41-3.07, p = 0.003)), whereas *Rickettsia* showed a weak but significant positive association with NDVI (β = 0.86, 95% CI = -0.03-1.60, p = 0.04). In contrast, *Staphylococcus* abundance was negatively associated with population density (β = -1.43, 95% CI = -2.83 to -0.27, p = 0.03) (Fig 2F). Collectively, these results demonstrate heterogeneous responses of different microbial taxa to environmental variables, highlighting the combined roles of human activity, vegetation cover, and livestock density in shaping the spatial distribution of tick-associated microbial communities [42–44].

### Pathogens and putative novel strain in *H. longicornis*

**Fig 3:**
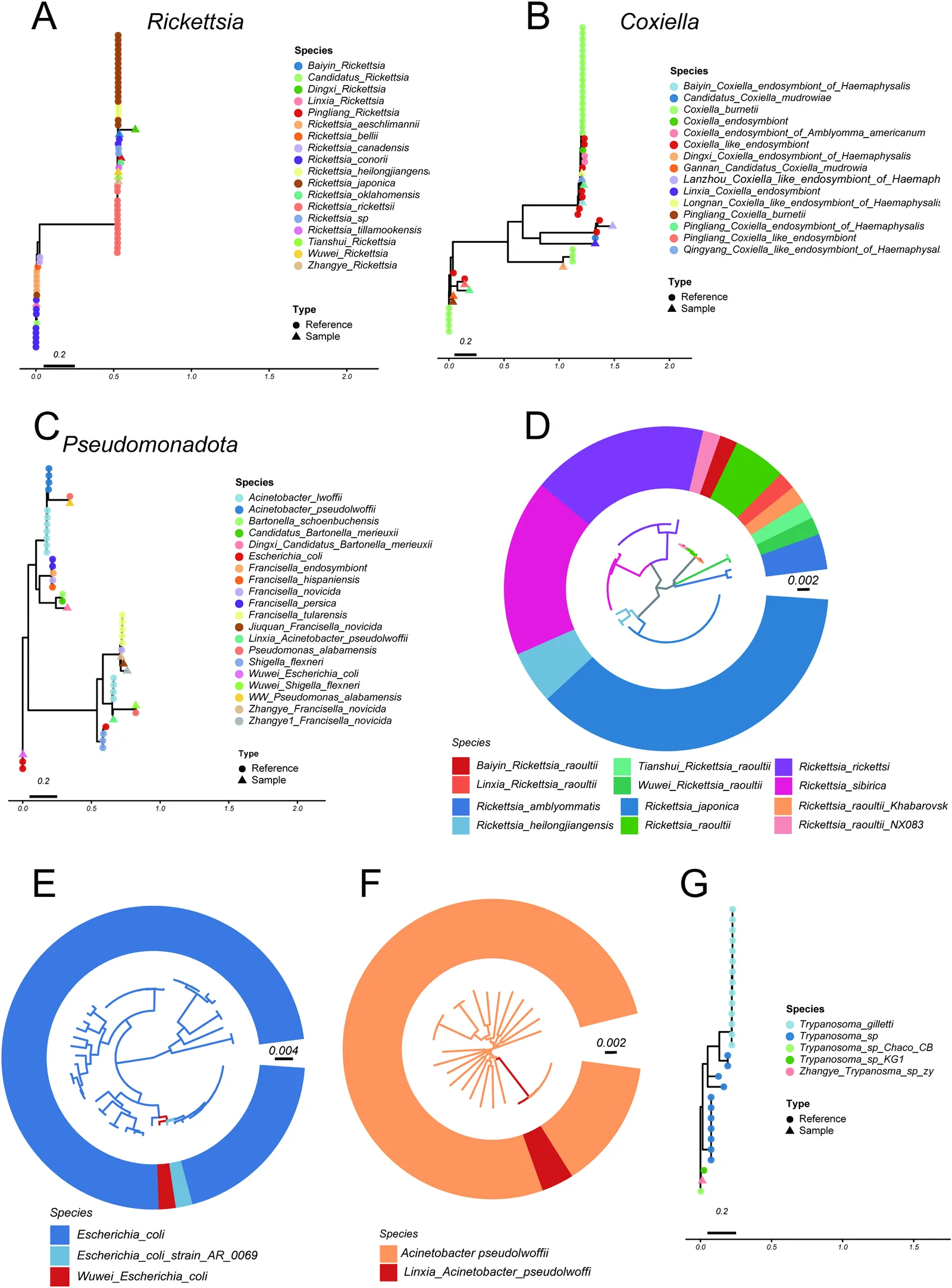
Maximum likelihood phylogenetic analyses based on 16S rRNA genes and SNP alignments. **(A)** *Rickettsia*, **(B)** *Coxiella*, and **(C)** *Pseudomonadota* inferred from 16S rRNA gene sequences. **(D)** *Rickettsia conorii* and *R. raoultii*, **(E)** *Escherichia coli*, and **(F)** *Acinetobacter pseudolwoffii* inferred from SNP alignments. **G)** *Trypanosoma* inferred from 18S rRNA gene sequences.

Through analysis of the 16S rRNA marker gene, we further confirmed and characterized the identified pathogens. In total, 12 pathogenic and opportunistic microorganisms were detected. For instance, *Rickettsia conorii* identified in Baiyin and Linxia, and *Rickettsia rickettsii* detected in Tianshui, Wuwei, and Zhangye (Fig 3A), are known to cause Boutonneuse fever and Rocky Mountain spotted fever, respectively [45]. *Coxiella burnetii*, the causative agent of Q fever [46], was identified in Pingliang and Dingxi (Fig 3B). The zoonotic pathogen *Francisella novicida*, associated with tularemia [47], was detected at low abundance in Jiuquan and Zhangye. In addition, *Escherichia coli* closely related to clinical isolates was identified in Wuwei.

Notably, several pathogens were reported for the first time in *H. longicornis* (Fig 3C). These include the zoonotic agent *Candidatus Bartonella merieuxii* [48] (detected in Dingxi), *Shigella flexneri* causing bacillary dysentery [49] (Wuwei), and opportunistic pathogens such as *Acinetobacter lwoffii* (Linxia) and *Moraxella atlantae* (Dingxi). Furthermore, *Pseudomonas alabamensis*, associated with plant diseases and bovine nasopharyngeal microbiota [50], and *Staphylococcus chromogenes*, a bovine mastitis pathogen [51], were detected in Wuwei.

Strain-level identification using StrainScan and SNP-based phylogenetic analysis revealed potential novel strains. The *Rickettsia raoultii* strains detected in Linxia and Baiyin showed high similarity to the Khabarovsk and NX083 reference strains, with average nucleotide identities (ANI) of 99.98% and 100%, respectively. In contrast, *Rickettsia conorii* strains from Tianshui and Wuwei exhibited lower ANI values (97.97% and 97.95%) and formed a distinct clade in the SNP phylogeny, suggesting the presence of novel strains. Similarly, the *Escherichia coli* strain (Fig 3E) identified in Wuwei (ANI = 99.76% to strain AR0069) and the *Acinetobacter pseudolwoffii-like* strain (Fig 3F) from Linxia (ANI = 99.79%) were also inferred as potential novel strains based on phylogenetic clustering [52]. Importantly, an 18S rRNA sequence closely related to *Trypanosoma* sp*. Chaco CB*, originally isolated from *Rhipicephalus microplus* ticks in Argentina, was identified in samples from Zhangye. This represents the first evidence of this pathogen in *H. longicornis* (Fig 3G).

These findings indicate that *H. longicornis* harbors a wide range of pathogenic and opportunistic microorganisms, including both well-established tick-borne pathogens and several taxa reported in this species for the first time. This expands our understanding of pathogen diversity in *H. longicornis* and its associated zoonotic risk.

### Spatial distribution and risk assessment of pathogens in *H. longicornis*

**Fig 4:**
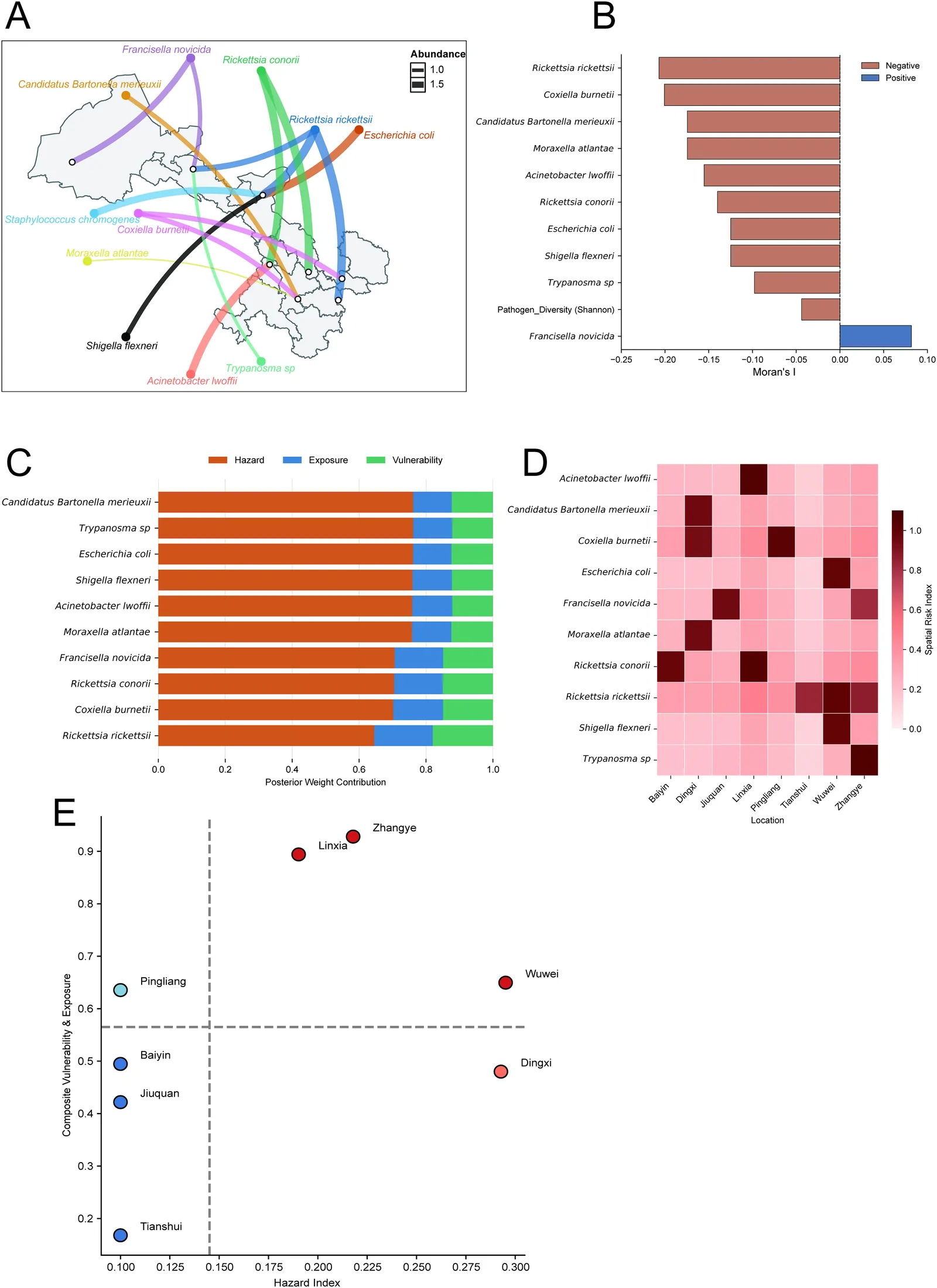
Spatial distribution and risk assessment of pathogens. **(A)** Network representation of pathogen distribution across regions. **(B)** Global spatial autocorrelation measured by Moran’s **I. (C)** Stacked bar chart showing the relative contributions of hazard, exposure, and vulnerability. **(D)** Heatmap illustrating the spatial distribution of pathogen-specific risk levels across eight regions. **(E)** Quadrant analysis of pathogen distribution patterns. **Map source**: Fig 4A was created by plotting our data on a basemap derived from the Natural Earth public-domain shapefiles. Direct link to the base layer data: https://www.naturalearthdata.com/downloads/10m-cultural-vectors/. Terms of use / license information: https://www.naturalearthdata.com/about/terms-of-use/).

We first assessed the spatial clustering of ten pathogens (Fig 4A) using global Moran’s I. Under the null hypothesis of complete spatial randomness, the expected value was E(I) ≈ −0.142. Overall, most pathogens did not exhibit significant global spatial autocorrelation, indicating predominantly random spatial distributions (Fig 4B). Specifically, the observed Moran’s I values for *Rickettsia conorii* (−0.140), *Escherichia coli* (−0.125), *Shigella flexneri* (−0.125), and *Acinetobacter lwoffii* (−0.155) were closely aligned with the theoretical expectation. In contrast, *Rickettsia rickettsii* (−0.206) and *Coxiella burnetii* (−0.200) showed a tendency toward spatial dispersion, whereas *Francisella novicida* (0.081) exhibited only weak positive spatial autocorrelation. Consistently, the Shannon diversity index of pathogens further supported this spatial independence, with an overall Moran’s I value of −0.043.

Consistent with the spatial autocorrelation analysis, the model identified hazard as the primary driver of the integrated spatial risk across pathogens. The mean weights of the hazard component ranged from 64.6% (*Rickettsia*) to 76.1% (*Moraxella atlantae*) (Fig 4C). In contrast, socio-ecological amplifiers, namely vulnerability and exposure, played important but secondary roles in modulating overall risk. For instance, the exposure and vulnerability components of Rickettsia-associated transmission risk exhibited the highest relative sensitivity, with weights reaching 17.4% and 17.9%, respectively. These findings indicate that spatial risk in the study area is primarily constrained by pathogen presence, while local socioeconomic heterogeneity−such as GDP, healthcare capacity, and livestock density−acts as a catalyst, amplifying the potential for localized risk expansion.

By integrating local driving factors with spatial multiplier effects, distinct epidemiological hotspots were identified (Fig 4D). Linxia emerged as a key high-risk node for specific pathogens, with the highest risk estimates observed for *Acinetobacter lwoffii* (mean risk: 1.04; 95% HDI: 0.96-1.18) and *Rickettsia conorii* (mean risk: 1.03; 95% HDI: 0.90-1.30). Wuwei and Zhangye were identified as major hotspots for multiple co-occurring pathogens. In Wuwei, the highest risk indices were observed for *Escherichia coli* (mean risk:0.99; 95% HDI: 0.88-1.13), *Rickettsia rickettsii* (mean risk:1.00; 95% HDI: 0.83-1.26), and *Shigella flexneri* (mean risk:0.99; 95% HDI: 0.87-1.12)). Meanwhile, Zhangye showed pronounced susceptibility to *Trypanosoma spp*. (mean risk: 1.05; 95% HDI: 0.97-1.18). Quadrant analysis further revealed marked heterogeneity in regional risk patterns (Fig 4E). Linxia, Zhangye, and Wuwei were located in the upper-right quadrant, characterized by high pathogen pressure coupled with strong environmental and socioeconomic amplification effects, and were therefore classified as high-risk hotspots.

### ARGs in the microbiome and MGEs

**Fig 5:**
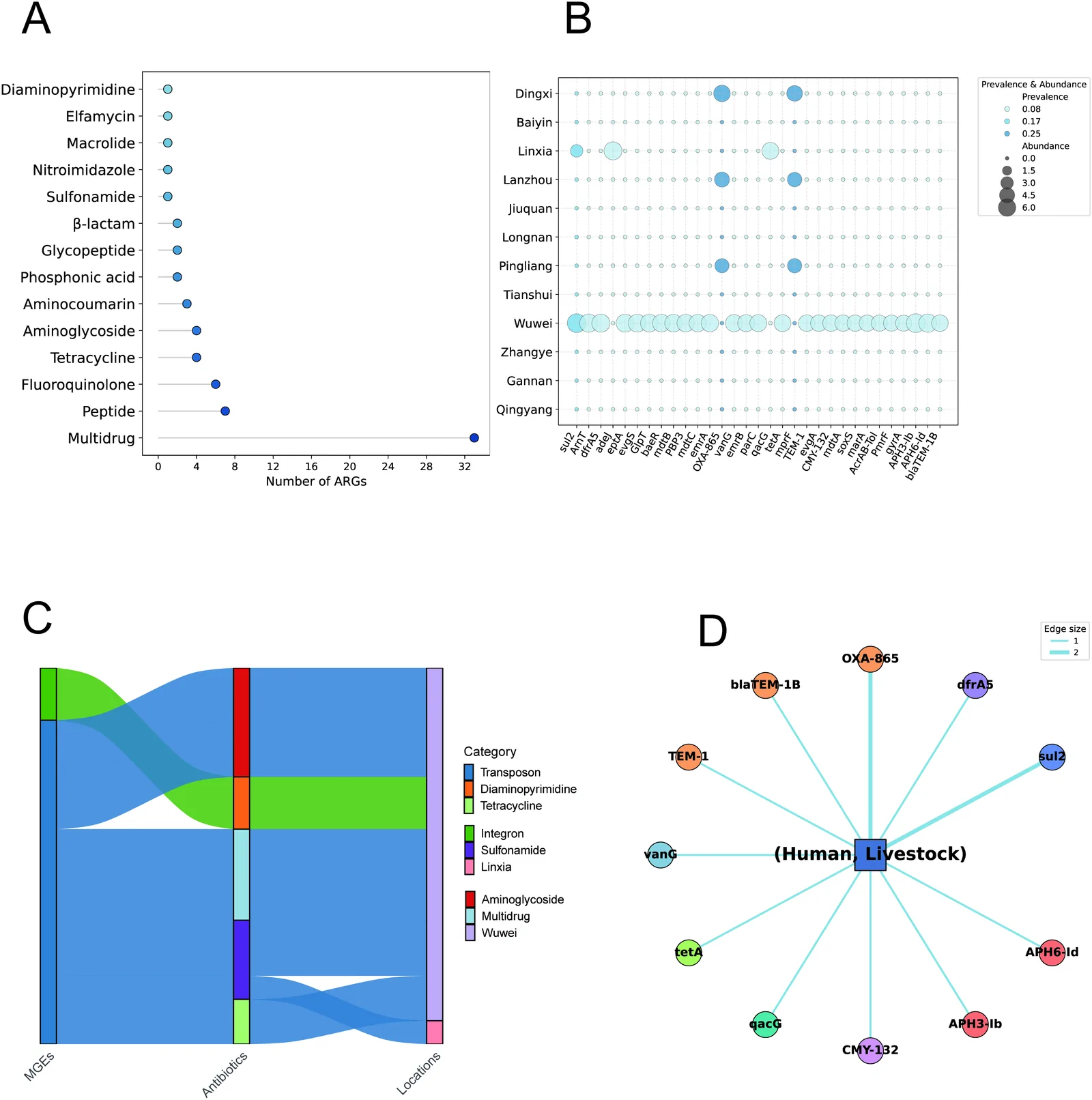
Antibiotic resistance genes(ARGs) and their associations with mobile genetic elements(MGEs). **(A)** Number of antibiotic classes represented by resistance genes detected in samples. **(B)** Abundance and prevalence of priority antibiotic resistance genes. The size and color of the circles represent their abundance and prevalence, respectively. **(C)** Co-occurrence patterns between antibiotic resistance genes and mobile genetic elements. **(D)** Antibiotic resistance genes in ticks showing close genetic similarity to those reported in human and livestock microbiomes.

The resistome of *H. longicornis* microbiomes was characterized by mapping open reading frames (ORFs) against the ResFinder database. From this, we identified 95 ARGs, which collectively confer resistance to 15 antibiotic classes (Fig 5A). To assess potential public health relevance, we defined “important ARGs” as resistance genes under enhanced surveillance, following established criteria [53]. Based on this classification, 31 priority ARGs were identified (Fig 5B), with the highest prevalence observed in samples from the Wuwei region. These priority ARGs were associated with resistance to multiple clinically important antibiotic classes, including aminoglycosides, sulfonamides,β-lactams, fluoroquinolones, tetracyclines, peptides, diaminopyrimidines, and nitroimidazoles. Within the tick-associated microbiome, several representative resistance genes commonly reported in human and livestock microbiomes were detected, including sul2, dfrA5, TEM-1, tetA, APH3-Ib, APH6-Id, and mdtF. There is high nucleotide-level similarity between these ARGs and corresponding resistance genes of human and livestock origin. Moreover, When ARG-MGE co-occurrence events were categorized by genetic context, approximately 90% were located on putative transposons, while 10% were found on integrons (Fig 5C). Notably, these ARGs not only shared high sequence similarity with resistance genes identified in human and livestock microbiomes but were also embedded within genetic contexts for MGE features (Fig 5D). These results suggest that *H. longicornis* may serve as a potential reservoir of antibiotic resistance genes associated with humans and livestock, and may play a contributory role in the dissemination of antimicrobial resistance.

## Discussion

In this study, we investigated the microbiome of *H. longicornis* collected from Gansu Province and identified a total of 25 bacterial genera and one protozoan genera. The phylum *Pseudomonadota* were dominant, which is consistent with previously reported characteristics of tick-associated microbial communities [54].The microbial inheritance in *H. longicornis* does not appear to be strictly deterministic but is likely influenced by both environmental filtering and stochastic processes. Environmental association analyses further revealed that the abundance of carried bacterial genera exhibited distinct ecological responses. Given that blood meals from different hosts may differentially influence tick microbiome diversity [55], further experimental studies involving controlled feeding on distinct animal hosts are warranted to ultimately evaluate the vector potential of *H. longicornis* across diverse ecological habitats. Because ticks were analyzed as pooled samples, the detected pathogen frequencies represent pool-level detection rather than individual-level prevalence. Consequently, the results should be interpreted as reflecting pathogen occurrence and diversity rather than precise estimates of infection prevalence. Collectively, these findings highlight the complex interplay among host-associated microbiota, environmental factors, and anthropogenic influences. Such heterogeneity is of critical importance for predicting pathogen transmission dynamics and for assessing region-specific public health risks.

Notably, *H. longicornis* harbors a highly diverse and complex microbial community, characterized by the co-occurrence of multiple tick-borne and other potentially pathogenic microorganisms [56,57]. Classical tick-borne pathogens, including *Rickettsia conorii*, *Rickettsia rickettsii*, *Coxiella burnetii*, and *Francisella novicida*, were consistently detected, indicating that this tick species maintains a substantial pathogen carriage potential in the study region. Importantly, this study reports for the first time the presence of several pathogens in *H. longicornis*, including *Candidatus Bartonella merieuxii*, *Acinetobacter lwoffii*, *Shigella flexneri*, *Staphylococcus chromogenes*, *Moraxella atlantae*, *Pseudomonas alabamensis*, and *Trypanosoma* [58]. In addition, putative novel strains were identified for *Rickettsia conorii*, *Escherichia coli*, and *Acinetobacter pseudolwoffii*. These findings substantially expand the known pathogen repertoire of the *H. longicornis* microbiome. *Candidatus Bartonella merieuxi*i, previously reported in the dog tick *Rhipicephalus sanguineus* [48], is considered a potential zoonotic pathogen. Its detection in *H. longicornis* suggests the existence of previously unrecognized transmission pathways, potentially involving cross-host transmission via tick bites or animal contact. The identification of putative novel strains further suggests that *H. longicornis* may serve as a natural reservoir or evolutionary melting pot for these pathogens. Genetic variation within these strains could influence pathogenicity, transmission efficiency, or antibiotic susceptibility. Future studies incorporating experimental infection models will be essential to validate the transmission competence and pathogenic potential of these microorganisms.

Spatial autocorrelation analysis indicated that the distribution of most pathogens detected in *H. longicornis* across the 12 regions was largely random. Consistent with this pattern, the integrated Bayesian risk model identified hazard-defined by pathogen presence and abundance-as the primary driver of spatial risk, with mean weights ranging from 64.6% to 76.1%. In contrast, socio-ecological amplifiers, namely vulnerability and exposure, played important but secondary roles in modulating overall risk. By delineating epidemiological hotspots, Linxia, Wuwei, and Zhangye were identified as high-risk regions. Although inter-regional spillover effects appeared limited, these findings highlight the need for highly targeted, location-specific, and pathogen-specific public health interventions, particularly in regions with elevated biological hazards and pronounced socio-ecological vulnerability. This integrated risk assessment framework supports a One Health approach that combines pathogen surveillance with socio-ecological risk stratification and may facilitate the development of more cost-effective intervention strategies [59].

From the tick microbiomes analyzed in this study, a total of 95 antibiotic resistance genes (ARGs) belonging to 15 antibiotic classes were identified. Of these, 31 were classified as clinically significant ARGs and exhibited particularly high prevalence in the Wuwei region. Representative ARGs, including sul2, TEM-1, and tetA, exhibited high sequence similarity to those commonly found in human and livestock sources [60,61]. Although direct evidence for the role of ticks in the transmission of antimicrobial resistance within human-animal-environment networks remains limited, our findings expand current knowledge of environmental resistome reservoirs and suggest that arthropod vectors warrant increased attention in future studies of antimicrobial resistance dissemination.

In summary, our findings confirm the diversity of pathogens harbored by *H. longicornis* and provide a comprehensive assessment of pathogen-associated risks in the study region. In addition, clinically relevant antibiotic resistance genes (ARGs) were identified within the tick microbiome. Together, these results offer valuable insights and resources for improving public health surveillance and guiding the prevention and control of risks associated with this tick species.

## Conclusion

This study provides a comprehensive metagenomic characterization of the microbial community associated with *H. longicornis* in Gansu Province, revealing substantial pathogen diversity and the presence of multiple antibiotic resistance genes. A total of 12 pathogenic microorganisms were identified, including several pathogens and putative novel strains reported in this species for the first time, thereby expanding the current understanding of the tick-borne pathogen spectrum.Bayesian risk assessment further identified Linxia, Zhangye, and Wuwei as key high-risk regions, offering valuable guidance for targeted surveillance and control strategies. Overall, these findings highlight the significant public health threat posed by *H. longicornis* and provide a foundation for future epidemiological monitoring and risk management efforts.

## Supporting information

S1 Table Numbers of ticks collected, sampling dates, geographic coordinates (latitude and longitude), and collection sites for each sampling location included in this study. (PDF)

## References

[1] Narvaez ZE, Egizi AM, Yabsley MJ, Thompson AT, Moustafa M, Alt E, et al. Multiple introductions of the Asian Longhorned Tick (*Haemaphysalis longicornis*) to the United States revealed using mitogenomics. Ecol Evol. 2025;15(4):e71312. doi: 10.1002/ece3.71312 40276246 PMC12018891

[2] Zhuang L, Du J, Cui X-M, Li H, Tang F, Zhang P-H, et al. Identification of tick-borne pathogen diversity by metagenomic analysis in *Haemaphysalis longicornis* from Xinyang, China. Infect Dis Poverty. 2018;7(1):45. doi: 10.1186/s40249-018-0417-4 29730989 PMC5937033

[3] Hoogstraal H, Roberts FH, Kohls GM, Tipton VJ. Review of *haemaphysalis (kaiseriana)* longicornis neumann (resurrected) of Australia, New Zealand, New Caledonia, Fiji, Japan, Korea, and Northeastern China and USSR, and its parthenogenetic and bisexual populations (Ixodoidea, Ixodidae). The Journal of Parasitology. 1968;:1197–213. 5757695

[4] Ronghang B, Roy B. Status of tick infections among semi-wild cattle in Arunachal Pradesh, India. Ann Parasitol. 2016;62(2):131–8. doi: 10.17420/ap6202.45 27614479

[5] Liu T, Feng X, Zhang Y, Liu J, Bao R. Genetic diversity of *Haemaphysalis longicornis* from China and molecular detection of Rickettsia. Exp Appl Acarol. 2019;79(2):221–31. doi: 10.1007/s10493-019-00423-y 31587112

[6] Fang L-Q, Liu K, Li X-L, Liang S, Yang Y, Yao H-W, et al. Emerging tick-borne infections in mainland China: An increasing public health threat. Lancet Infect Dis. 2015;15(12):1467–79. doi: 10.1016/S1473-3099(15)00177-2 26453241 PMC4870934

[7] Zhao L, Li J, Cui X, Jia N, Wei J, Xia L, et al. Distribution of Haemaphysalis longicornis and associated pathogens: Analysis of pooled data from a China field survey and global published data. Lancet Planet Health. 2020;4(8):e320–9. doi: 10.1016/S2542-5196(20)30145-5 32800150

[8] Thompson AT, White SA, Shaw D, Garrett KB, Wyckoff ST, Doub EE, et al. A multi-seasonal study investigating the phenology, host and habitat associations, and pathogens of Haemaphysalis longicornis in Virginia, U.S.A. Ticks Tick Borne Dis. 2021;12(5):101773. doi: 10.1016/j.ttbdis.2021.101773 34229999

[9] Ni X-B, Cui X-M, Liu J-Y, Ye R-Z, Wu Y-Q, Jiang J-F, et al. Metavirome of 31 tick species provides a compendium of 1,801 RNA virus genomes. Nat Microbiol. 2023;8(1):162–73. doi: 10.1038/s41564-022-01275-w 36604510 PMC9816062

[10] Tufts DM, Sameroff S, Tagliafierro T, Jain K, Oleynik A, VanAcker MC, et al. A metagenomic examination of the pathobiome of the invasive tick species, *Haemaphysalis longicornis*, collected from a New York City borough, USA. Ticks Tick Borne Dis. 2020;11(6):101516. doi: 10.1016/j.ttbdis.2020.101516 32993936

[11] Cumbie AN, Trimble RN, Eastwood G. Pathogen spillover to an invasive tick species: First detection of bourbon virus in *Haemaphysalis longicornis* in the United States. Pathogens. 2022;11(4):454. doi: 10.3390/pathogens11040454 35456129 PMC9030182

[12] Price KJ, Ayres BN, Maes SE, Witmier BJ, Chapman HA, Coder BL, et al. First detection of human pathogenic variant of Anaplasma phagocytophilum in field-collected *Haemaphysalis longicornis*, Pennsylvania, USA. Zoonoses Public Health. 2022;69(2):143–8. doi: 10.1111/zph.12901 34958171

[13] Price KJ, Graham CB, Witmier BJ, Chapman HA, Coder BL, Boyer CN. Borrelia burgdorferi sensu stricto DNA in field-collected *Haemaphysalis longicornis* ticks, Pennsylvania, United States. Emerging Infectious Diseases. 2021;27(2):608. 10.3201/eid2702.201552 PMC7853548 33496234

[14] Thompson AT, White S, Shaw D, Egizi A, Lahmers K, Ruder MG. Theileria orientalis Ikeda in host-seeking *Haemaphysalis longicornis* in Virginia, USA. Ticks and tick-borne diseases. 2020;11(5):101450. 10.1016/j.ttbdis.2020.101450 32723633

[15] Wu Y, Sun Y, Liu J, Ma Y, Fang L, Zhang Y, et al. Ticks carry various antibiotic resistance genes and can serve as vectors for their dissemination and as reservoirs by vertical propagation. Environ Res. 2024;262(Pt 2):119976. doi: 10.1016/j.envres.2024.119976 39270953

[16] Ma R, Shi Y, Wu W, Huang C, Xue F, Hou R, et al. The bacterial diversity and potential pathogenic risks of giant panda-infesting ticks. Microbiol Spectr. 2025;13(7):e0219724. doi: 10.1128/spectrum.02197-24 40494644 PMC12211003

[17] Zhang J, Liu X, Qin Y, Fan Y, Cheng S. Wetlands mapping and monitoring with long-term time series satellite data based on google earth engine, random forest, and feature optimization: A case study in Gansu Province, China. Land. 2024;13(9):1527. doi: 10.3390/land13091527

[18] Chen Z, Yang X, Bu F, Yang X, Yang X, Liu J. Ticks (acari: ixodoidea: argasidae, ixodidae) of China. Exp Appl Acarol. 2010;51(4):393–404. doi: 10.1007/s10493-010-9335-2 20101443

[19] Chen S, Zhou Y, Chen Y, Gu J. fastp: An ultra-fast all-in-one FASTQ preprocessor. Bioinformatics. 2018;34(17):i884–90. doi: 10.1093/bioinformatics/bty560 30423086 PMC6129281

[20] Chuvochina M, Gerken J, Frentrup M, Sandikci Y, Goldmann R, Freese HM, et al. SILVA in 2026: A global core biodata resource for rRNA within the DSMZ digital diversity. Nucleic Acids Res. 2026;54(D1):D334–41. doi: 10.1093/nar/gkaf1247 41251139 PMC12807666

[21] Langmead B, Salzberg SL. Fast gapped-read alignment with Bowtie 2. Nat Methods. 2012;9(4):357–9. doi: 10.1038/nmeth.1923 22388286 PMC3322381

[22] Li D, Liu C-M, Luo R, Sadakane K, Lam T-W. MEGAHIT: an ultra-fast single-node solution for large and complex metagenomics assembly via succinct de Bruijn graph. Bioinformatics. 2015;31(10):1674–6. doi: 10.1093/bioinformatics/btv033 25609793

[23] Gemayel K, Lomsadze A, Borodovsky M. MetaGeneMark-2: Improved gene prediction in metagenomes. BioRxiv. 2022. doi: 10.1101/2022.07.25.500264

[24] Li W, Godzik A. Cd-hit: A fast program for clustering and comparing large sets of protein or nucleotide sequences. Bioinformatics. 2006;22(13):1658–9. doi: 10.1093/bioinformatics/btl158 16731699

[25] Buchfink B, Xie C, Huson DH. Fast and sensitive protein alignment using DIAMOND. Nat Methods. 2015;12(1):59–60. doi: 10.1038/nmeth.3176 25402007

[26] Huson DH, Auch AF, Qi J, Schuster SC. MEGAN analysis of metagenomic data. Genome Res. 2007;17(3):377–86. doi: 10.1101/gr.5969107 17255551 PMC1800929

[27] Danecek P, Bonfield JK, Liddle J, Marshall J, Ohan V, Pollard MO, et al. Twelve years of SAMtools and BCFtools. Gigascience. 2021;10(2):giab008. doi: 10.1093/gigascience/giab008 33590861 PMC7931819

[28] Nguyen L-T, Schmidt HA, von Haeseler A, Minh BQ. IQ-TREE: A fast and effective stochastic algorithm for estimating maximum-likelihood phylogenies. Mol Biol Evol. 2015;32(1):268–74. doi: 10.1093/molbev/msu300 25371430 PMC4271533

[29] Liao H, Ji Y, Sun Y. High-resolution strain-level microbiome composition analysis from short reads. Microbiome. 2023;11(1):183. doi: 10.1186/s40168-023-01615-w 37587527 PMC10433603

[30] Jain C, Rodriguez-R LM, Phillippy AM, Konstantinidis KT, Aluru S. High throughput ANI analysis of 90K prokaryotic genomes reveals clear species boundaries. Nat Commun. 2018;9(1):5114. doi: 10.1038/s41467-018-07641-9 30504855 PMC6269478

[31] Treangen TJ, Ondov BD, Koren S, Phillippy AM. The Harvest suite for rapid core-genome alignment and visualization of thousands of intraspecific microbial genomes. Genome Biol. 2014;15(11):524. doi: 10.1186/s13059-014-0524-x 25410596 PMC4262987

[32] Hunter JD. Matplotlib: A 2D Graphics Environment. Comput Sci Eng. 2007;9(3):90–5. doi: 10.1109/mcse.2007.55

[33] Pedregosa F, Varoquaux G, Gramfort A, Michel V, Thirion B, Grisel O. Scikit-learn: Machine learning in Python. The Journal of Machine Learning Research. 2011;12:2825–30.

[34] Sánchez CA, Li H, Phelps KL, Zambrana-Torrelio C, Wang L-F, Zhou P, et al. A strategy to assess spillover risk of bat SARS-related coronaviruses in Southeast Asia. Nat Commun. 2022;13(1):4380. doi: 10.1038/s41467-022-31860-w 35945197 PMC9363439

[35] Hosseini PR, Mills JN, Prieur-Richard A-H, Ezenwa VO, Bailly X, Rizzoli A, et al. Does the impact of biodiversity differ between emerging and endemic pathogens? The need to separate the concepts of hazard and risk. Philos Trans R Soc Lond B Biol Sci. 2017;372(1722):20160129. doi: 10.1098/rstb.2016.0129 28438918 PMC5413877

[36] Hyatt D, Chen G-L, Locascio PF, Land ML, Larimer FW, Hauser LJ. Prodigal: Prokaryotic gene recognition and translation initiation site identification. BMC Bioinformatics. 2010;11:119. doi: 10.1186/1471-2105-11-119 20211023 PMC2848648

[37] Bortolaia V, Kaas RS, Ruppe E, Roberts MC, Schwarz S, Cattoir V, et al. ResFinder 4.0 for predictions of phenotypes from genotypes. J Antimicrob Chemother. 2020;75(12):3491–500. doi: 10.1093/jac/dkaa345 32780112 PMC7662176

[38] Madden T. The BLAST sequence analysis tool. The NCBI handbook. 2013. pp. 425–36.

[39] Siguier P, Perochon J, Lestrade L, Mahillon J, Chandler M. ISfinder: The reference centre for bacterial insertion sequences. Nucleic Acids Res. 2006;34(Database issue):D32-6. doi: 10.1093/nar/gkj014 16381877 PMC1347377

[40] Ross K, Varani AM, Snesrud E, Huang H, Alvarenga DO, Zhang J, et al. TnCentral: A Prokaryotic Transposable Element Database and Web Portal for Transposon Analysis. mBio. 2021;12(5):e0206021. doi: 10.1128/mBio.02060-21 34517763 PMC8546635

[41] Moura A, Soares M, Pereira C, Leitão N, Henriques I, Correia A. INTEGRALL: A database and search engine for integrons, integrases and gene cassettes. Bioinformatics. 2009;25(8):1096–8. doi: 10.1093/bioinformatics/btp105 19228805

[42] Vanroy T, Martel A, Baeten L, Fonville M, Lens L, Pasmans F, et al. The effect of forest structural complexity on tick-borne pathogens in questing ticks and small mammals. Forest Ecology and Management. 2024;562:121944. doi: 10.1016/j.foreco.2024.121944

[43] Rataud A, Drouin A, Bournez L, Pisanu B, Moutailler S, Henry P-Y, et al. Contributions of birds to the feeding of ticks at host community level: Effects of tick burden, host density and yearly fluctuations. Ticks Tick Borne Dis. 2024;15(6):102390. doi: 10.1016/j.ttbdis.2024.102390 39241452

[44] Ye R-Z, Li Y-Y, Xu D-L, Wang B-H, Wang X-Y, Zhang M-Z, et al. Virome diversity shaped by genetic evolution and ecological landscape of Haemaphysalis longicornis. Microbiome. 2024;12(1):35. doi: 10.1186/s40168-024-01753-9 38378577 PMC10880243

[45] Maggi RG, Krämer F. A review on the occurrence of companion vector-borne diseases in pet animals in Latin America. Parasit Vectors. 2019;12(1):145. doi: 10.1186/s13071-019-3407-x 30917860 PMC6438007

[46] Eldin C, Mélenotte C, Mediannikov O, Ghigo E, Million M, Edouard S, et al. From Q Fever to *Coxiella burnetii* Infection: A Paradigm Change. Clin Microbiol Rev. 2017;30(1):115–90. doi: 10.1128/CMR.00045-16 27856520 PMC5217791

[47] Kingry LC, Petersen JM. Comparative review of *Francisella tularensis* and *Francisella novicida*. Front Cell Infect Microbiol. 2014;4:35. doi: 10.3389/fcimb.2014.00035 24660164 PMC3952080

[48] Chomel BB, McMillan-Cole AC, Kasten RW, Stuckey MJ, Sato S, Maruyama S. Candidatus Bartonella merieuxii, a potential new zoonotic Bartonella species in canids from Iraq. 2012. 10.1371/journal.pntd.0001843 PMC3459848 23029597

[49] Thompson CN, Duy PT, Baker S. The rising dominance of *Shigella sonnei*: An intercontinental shift in the etiology of bacillary dysentery. PLoS Negl Trop Dis. 2015;9(6):e0003708. doi: 10.1371/journal.pntd.0003708 26068698 PMC4466244

[50] Fullem KR, MacLellan MP, Iriarte FB, Poudel M, Capik S, Dedonder K, et al. From cantaloupe to cattle: Pseudomonas alabamensis sp. nov. described from diseased cantaloupe (Cucumis melo) foliage and a bovine (Bos taurus) nasopharynx. Int J Syst Evol Microbiol. 2025;75(7):006848. doi: 10.1099/ijsem.0.006848 40658455 PMC12281984

[51] Simojoki H, Orro T, Taponen S, Pyörälä S. Host response in bovine mastitis experimentally induced with Staphylococcus chromogenes. Vet Microbiol. 2009;134(1–2):95–9. doi: 10.1016/j.vetmic.2008.09.003 18938048

[52] Rodriguez-R LM, Conrad RE, Viver T, Feistel DJ, Lindner BG, Venter SN, et al. An ANI gap within bacterial species that advances the definitions of intra-species units. mBio. 2024;15(1):e0269623. doi: 10.1128/mbio.02696-23 38085031 PMC10790751

[53] Shi Y, Li Y, Li H, Haerheng A, Marcelino VR, Lu M, et al. Extensive cross-species transmission of pathogens and antibiotic resistance genes in mammals neglected by public health surveillance. Cell. 2025;188(23):6591-6605.e14. doi: 10.1016/j.cell.2025.08.016 40865528

[54] Grandi G, Chiappa G, Ullman K, Lindgren P-E, Olivieri E, Sassera D, et al. Characterization of the bacterial microbiome of Swedish ticks through 16S rRNA amplicon sequencing of whole ticks and of individual tick organs. Parasit Vectors. 2023;16(1):39. doi: 10.1186/s13071-022-05638-4 36717919 PMC9885626

[55] Swei A, Kwan JY. Tick microbiome and pathogen acquisition altered by host blood meal. ISME J. 2017;11(3):813–6. doi: 10.1038/ismej.2016.152 27858931 PMC5322304

[56] Perveen N, Muzaffar SB, Vijayan R, Al-Deeb MA. Microbial communities associated with the camel tick, Hyalomma dromedarii: 16S rRNA gene-based analysis. Sci Rep. 2020;10(1):17035. doi: 10.1038/s41598-020-74116-7 33046763 PMC7550333

[57] Boularias G, Azzag N, Galon C, Šimo L, Boulouis H-J, Moutailler S. High-throughput microfluidic real-time PCR for the detection of multiple microorganisms in ixodid cattle ticks in Northeast Algeria. Pathogens. 2021;10(3):362. doi: 10.3390/pathogens10030362 33803682 PMC8002991

[58] Morel N, Thompson CS, Rossner MV, Mangold AJ, Nava S. A trypanosoma species detected in Rhipicephalus (Boophilus) microplus ticks from Argentina. Ticks and tick-borne diseases. 2021;12(1):101573. 10.1016/j.ttbdis.2020.101573 33007666

[59] Dantas-Torres F, Chomel BB, Otranto D. Ticks and tick-borne diseases: A One Health perspective. Trends Parasitol. 2012;28(10):437–46. doi: 10.1016/j.pt.2012.07.003 22902521

[60] Wei N, Lu J, Dong Y, Li S. Profiles of microbial community and antibiotic resistome in wild tick species. mSystems. 2022;7(4):e0003722. doi: 10.1128/msystems.00037-22 35913190 PMC9426550

[61] Chavarría-Bencomo IV, Nevárez-Moorillón GV, Espino-Solís GP, Adame-Gallegos JR. Antibiotic resistance in tick-borne bacteria: A one health approach perspective. J Infect Public Health. 2023;16 Suppl 1:153–62. doi: 10.1016/j.jiph.2023.10.027 37945496

