# Supplementary material for: Metagenomic insights into pathogen diversity and public health risk of Haemaphysalis longicornis in Gansu Province of China
Source: PLoS Negl Trop Dis. 2026 Sep 15;20(9):e0014713. doi: 10.1371/journal.pntd.0014713 (PMC13588528; doi:10.1371/journal.pntd.0014713)
Supplement: Supplementary file 1 [file pntd.0014713.s001.pdf]

Supplementary Table S1

Location	No. of ticks	Collection date	Longitude	Latitude
Dingxi	70	2025/4/29	104.57	34.72
Lanzhou	20	2025/4/9	104.11	35.84
Pingliang	60	2025/4/23	106.57	35.45
Wuwei	20	2025/4/23	103.09	38.62
Linxia	20	2025/6/3	103.29	35.96
Tianshui	20	2025/4/16	106.4	34.68
Zhangye	30	2025/5/30	99.82	39.38
Gannan	10	2025/4/1	102.91	34.98
Baiyin	10	2025/5/1	105.05	35.69
Jiuquan	10	2025/5/29	94.34	39.63
Qingyang	10	2025/4/26	107.64	35.71
Longnan	10	2025/5/5	105.61	33.33
